# Staphylococcal enterotoxins in the Etiopathogenesis of Mucosal Autoimmunity within the Gastrointestinal Tract

**DOI:** 10.3390/toxins6051471

**Published:** 2014-04-25

**Authors:** MaryAnn Principato, Bi-Feng Qian

**Affiliations:** 1 Division of Toxicology, Office of Applied Research and Safety Assessment, Center for Food Safety and Applied Nutrition, US Food and Drug Administration, 8301 Muirkirk Road, Laurel, MD 20708, USA; 2 Commissioner’s Fellowship Program, Division of Toxicology, Office of Applied Research and Safety Assessment, Center for Food Safety and Applied Nutrition, US Food and Drug Administration, 8301 Muirkirk Road, Laurel, MD 20708, USA; E-Mail: bifeng.qian@fda.hhs.gov

**Keywords:** Staphylococcal enterotoxin B, superantigen, food poisoning, Treg, gut-associated lymphoid tissue, intestinal inflammation, *Staphylococcus aureus*

## Abstract

The staphylococcal enterotoxins (SEs) are the products of *Staphylococcus aureus* and are recognized as the causative agents of classical food poisoning in humans following the consumption of contaminated food. While illness evoked by ingestion of the SE or its producer organism in tainted food are often self-limited, our current understanding regarding the evolution of *S. aureus* provokes the utmost concern. The organism and its associated toxins, has been implicated in a wide variety of disease states including infections of the skin, heart, sinuses, inflammatory gastrointestinal disease, toxic shock, and Sudden Infant Death Syndrome. The intricate relationship between the various subsets of immunocompetent T cells and accessory cells and the ingested material found within the gastrointestinal tract present daunting challenges to the maintenance of immunologic homeostasis. Dysregulation of the intricate balances within this environment has the potential for extreme consequences within the host, some of which are long-lived. The focus of this review is to evaluate the relevance of staphylococcal enterotoxin in the context of mucosal immunity, and the underlying mechanisms that contribute to the pathogenesis of gastrointestinal autoimmune disease.

## 1. The Staphylococcal enterotoxins and Disease

The staphylococcal enterotoxins (SEs), produced by *Staphylococcus aureus*, are recognized as etiologic agents of food poisoning in man, and as potent immunologic superantigens, that produce clinically important syndromes based on their capacity to induce T cell activation and proliferation that lead to the production of significant quantities of proinflammatory mediators. Early recognition of their characteristic as superantigens promoted theoretical considerations that these microbial contaminants might contribute to the underlying causes of self-reactive autoimmune disease due to the nature of the interaction between the microbial superantigen and host cells [1,2]. The SEs have since been implicated in the induction of several human autoimmune exacerbations that include allergic airway disease [3,4], atopic dermatitis [5,6], inflammatory arthritic conditions [3], and colitis [7]. Indeed, patients exhibiting dust mite-induced allergic rhinitis or asthma demonstrate significant levels of IgE antibodies to SEA, SEB, and SEC [8] as well as elevated levels of serum eosinophil cationic protein, an indicator of asthma and rhinitis. More recently, a murine model demonstrated that primary dermal sensitization with both peanut extract and SEB, supported a Th2-mediated IL-4 dependent secondary response to peanut extract, presenting a possible mechanism for the induction of some food allergies [9]. Taken together, the superantigenic staphylococcal enterotoxins provide multiple mechanisms for the induction of organ-specific and systemic autoimmune disease, in addition to classic pathogenic states that includes food poisoning. 

The staphylococcal enterotoxins (SEs) have long been implicated in food-borne diseases, and are produced by enterotoxigenic strains of the pathogen *Staphylococcus aureus* [10,11]. Several enterotoxigenic toxins are attributed to *S. aureus* which include the classic serotypes SEA, SEB, SEC, SED, and SEE [12,13]. Molecular techniques have revealed additional closely related enterotoxins including SEG, SEH [14,15], SEI, SEJ, SEK, SEL, SEM [16,17], SEN, SEO, SEP, SER [18,19] and SEU [18,20]. These products have been identified following isolation from food samples implicated in food poisoning scenarios and clinical samples derived from affected individuals [16,18,19,21,22,23,24,25]. So far, more than 20 different types of SEs and enterotoxin –like molecules (SEl) have been described (SEA-SEIV) that share phylogenetic relationship, structure, and sequence similarities [26,27]. It should be noted that while several of the newly identified enterotoxins were identified in various contamination scenarios, it is not yet known whether all of the identified enterotoxins directly induce emetic disease in humans. Further, their possible role in the development or induction of autoimmune disease has not been described. These toxic proteins are composed of approximately 220–240 amino acids and demonstrate an averaged molecular size of 25 kD. They are encoded on plasmids, bacteriophages, pathogenicity islands, and mobile genetic elements [28,29], and demonstrate a remarkable conservation of structural architecture [30,31].

Surveillance of food-borne illness by the Centers for Disease Control and Prevention (CDC) has revealed that more than 200 known diseases are transmitted through food, which cause approximately 76 million cases, 325,000 hospitalizations, and 5000 deaths in the United States annually [32]. However, because many cases of food-borne diseases may not seek medical attention or come to the notice of public health authorities, it is likely that the true incidence is significantly greater than that estimated from investigations of suspected food-borne disease outbreaks. Among the bacteria that can cause food-borne illness, *Staphylococcus aureus* is particularly noteworthy due to the ability to produce toxins that are etiologic agents of gastroenteritis, implicated in autoimmune dysregulation, and lack any FDA-approved therapeutic measures or vaccines to counter the organism or its products. *Staphylococcus aureus* has been most often implicated in staphylococcal food poisoning which is usually attributed to the improper handling of food. It is an encapsulated Gram-positive facultative anaerobic bacteria whose pathogenicity is supported by its multiple virulence factors that include leukocidins, hemolysins, antibiotic resistance(s), exfoliative toxins, toxic shock syndrome toxin-1 (TSST1), and multiple homologous enterotoxins. The bacterium is linked to food poisoning and multiple human illnesses such as endocarditis and pneumonia, and skin infections including toxic shock boils, cellulitis, scalded skin syndrome, and has been implicated in Sudden Infant Death Syndrome [33,34]. Up to 30%–50% of healthy people can harbor the bacteria in the nostrils, on skin, and on hair [22,35]. Interestingly, it is only in a small proportion of the food poisoning outbreaks that the same strain of disease-causing bacteria has been identified from the food handler, food and/or the victim [36,37]. The bacteria may maintain steady growth in a wide range of temperatures, pHs, and sodium chloride concentrations [38]. These characteristics enable the bacteria to contaminate and spread in a great variety of food, including foods that require extensive manipulation during processing. Indeed, the SEB toxin in particular has been characterized as being particularly resistant to destruction by heating [25,39] and is able to withstand storage in acidic foods [40]. The ingested dose of SE required to induce illness in humans has not been determined. Early volunteer studies reported that administration of 50 ug [41] into volunteers weighing 145 lbs and less induced illness. However nanogram quantities have been estimated to induce illness [24,42], and the degree of severity can vary with individual sensitivity. In a large US outbreak caused by ingestion of SEA-contaminated chocolate milk [24], the mean concentration of the toxin in a 400-mL container was approximately 0.5 ng/mL. The usual incubation period of staphylococcal food poisoning is between 2 and 6 h [41], depending on the amount of toxin ingested. Typical disease is characterized by cramping abdominal pain, nausea, vomiting, sometimes followed by diarrhea. Physical examination may reveal dehydration and hypotension if fluid loss has been significant. Routine laboratory testing may detect an electrolyte disturbance. Approximately 10% of the individuals with the food-borne disease need to be hospitalized for further assessment and management. Mortality is rare as demonstrated by a study on 7126 patients where the case fatality rate was as low as 0.03% and the death was exclusively seen in the elderly group [36]. Several mechanisms have been proposed to explain how SEs cause enteric illness: (1) the release of proinflammatory cytokines as a result of SE-induced superantigenic T cell proliferation; (2) the binding of SEs to intestinal mast cells that leads to degranulation [43,44]; and (3) a direct effect upon the intestinal epithelium affecting gut transit [38]. Elegant studies using cultured porcine jejunal sections have demonstrated the binding of SEA and SEB toxins onto the surface of the enterocyte microvillus, possibly mediated by binding onto digalactosylceramide residues. This research further demonstrated that the toxins appear within sub-apical punctae, indicative of entry into the enterocytes via apical endocytosis within the endosomes [45]. It should be noted however, that the pathological events following SE introduction into the intestinal area have been shown to be due to the combined effect of SE toxins and toxins that disrupt the epithelial barrier by inducing enterocyte-cytopathic toxins produced by *Staphylococcus aureus* [46]. In addition, a 5-HT or serotonin-mediated pathway has been reported recently [47].

## 2. Immunity within the Gut Associated Lymphoid Tissue

The gastrointestinal tract is typically characterized by a large surface area that is responsible for the digestion and absorption of ingested nutrients. This function is aided by the intestine’s mucosal lining, whose absorptive surface is increased by the presence of villi which are composed of a single layer of epithelial cells containing a rich network of capillaries and lymphatics and project into the lumen. The intestinal lumen normally contains ingested material consisting of degraded dietary products, commensal microbial flora, and any ingested contaminants including pathogenic bacteria and their products, viruses, fungi, or parasites. Thus, absorption of essential nutrients and host immune defense, two apparently divergent processes, must occur in the intestinal mucosa. In order to provide a healthy microenvironment for the normal physiological activities of the gut, the immune system within the gut-associated lymphoid tissue (GALT) must: (1) generate immunologic tolerance towards nutrients and the commensal microflora; and (2) recognize and abolish infectious agents and potentially injurious toxins [48,49,50]. 

Beneath the epithelial layer of the mammalian gastrointestinal tract lies a rich source of immunocompetent cells that comprise a significant portion of the body’s T cells. While the peripheral immune system contains effector T lineage cells bearing the αβ T cell receptor (TCR) which are composed of either class II-restricted CD4^+^ T cells or class I-restricted CD8^+^T cells, the intraepithelial lymphocytes are distinguished by the predominant presence of homodimeric CD8αα^+^ T cells and T lineage cells containing the γδTCR [51]. The γδ-T cell enriched Intraepithelial lymphocytes (IEL) function as a surveillance system for damaged or infected epithelial cells, and may modulate local immune responses by controlling cellular traffic and limiting mucosal access of inflammatory cells. Substantial numbers of αβTCR T cells are present in lamina propria of the gastrointestinal tract that display an activated/memory phenotype consisting mainly of MHC class II-restricted CD4^+^ T helper (Th) cells. However, the intestinal mucosa harbors all of the major Th-cell subsets (Th1, Th2, Th17, and Tfh) that are defined by their lineage-specific transcription factor expression, cytokine production, and subsequent immune function. Additionally, redundant regulatory strategies include naturally occurring and adaptive CD4^+^CD25^+^Foxp3^+^ regulatory T cells. A functionally specialized population of CD103^+^ dendritic cells that are enriched in the lamina propria of intestine and mesenteric lymph nodes (MLN) are highly effective in promoting the conversion of naive CD4^+^ T cells into Foxp3^+^ T cells in an antigen-specific manner and in maintaining the stability of preexisting Foxp3^+^ cell population [52,53]. Foxp3^+^ Treg cells are pivotal in the control of intestinal homeostasis and their action is achieved via a dual mechanism involving direct cell-to-cell interactions or the release of regulatory cytokines, TGF-β and IL-10 [54,55,56]. It is worthy of note that mice orally tolerized to a specific antigen may produce a great number of converted Foxp3^+^ T cells in the Peyer’s Patches (PP), lamina propria, and mesenteric lymph nodes [53]. 

The innate immune cells of the intestinal mucosa includes macrophages, dendritic cells, the M cells found on the dome epithelium of Peyer’s Patches and recognized for their endocytic activity, that are involved in critical activities pertaining to the initiation of pathogen defense, maintenance of tissue homeostasis [57,58,59]. Gastrointestinal macrophages are distinguished by possessing cell surface Fc receptors that bind the Fc portion of IgG immunoglobulin, complement C3b and C3d receptors, MHC Class I and Class II, Toll like receptors (TLR) [60,61], the F4/80 marker [62], cytokine receptors and the CX_3_CR1 receptor for fractalkine (CX3CL1). The chemokine receptor CX_3_CR1 is widely expressed by macrophages, dendritic cells, T cells, and intestinal epithelial cells. Importantly, dendritic cells are involved in the control of Treg development and function in the gut. Research by Neiss and coworkers has demonstrated the critical importance of CX_3_CR1^+^ dendritic cells (DC) in the surveillance and defense against pathogens since these cells can directly sample the intestinal lumen by use of their transepithelial dendrites [63], representing a pathway that is distinct from previously established pathways of antigen transit from the gastrointestinal lumen involving the specialized M cells of the epithelial layer. 

During a gastrointestinal immune response, ingested antigens in the lumen enter the Peyer’s Patches via the specialized epithelial cells known as M cells present in the epithelial layer overlaying the PP. The M cells endocytose antigen, effectively transporting the antigen into the interior of the PP where dendritic cells and phagocytic cells process the antigen via intracellular metabolic and proteolytic degradation and modification. The resulting fragment [58,64], is transported to the surface of the cell where it is presented in conjunction with the major histocompatibility (MHC) gene molecule. The presentation of antigen is critical for the activation of the appropriate responding T cell, which contains a great variability of gene sequences, which must be rearranged to configure a mature, functional, TCR. This permits the specific recognition of the presented peptide sequence by the TCR of the responding T cell, and provides for the development of the adaptive immune response, initiating T cell activation and differentiation into antigen-specific effector cells. Antigen-specific FoxP3^+^CD4^+^ are critical for the control of effector responses in an antigen specific manner [65]. Following activation, the activated T cells migrate to the gut lamina propria effector site via an appropriate homing mechanism mediated by addressins-integrins [66]. The route of antigen sampling, the form and concentration of antigen, and the host’s immunologic status (age, previous sensitizations) will determine the course of immune responsiveness [67]. 

### Superantigenic Response in the Gastrointestinal Mucosa

In contrast, an immune response driven by a superantigen presents the scenario in which the requirements for antigen internalization, processing, and expression are circumvented due to the characteristic binding of this class of molecule. The defining characteristic of a superantigen is its unconventional binding to the MHC class II molecules, and to non-antigen-specific sequences found on the β chain of the T cell receptors (TCR) [68] of humans and mice. The staphylococcal enterotoxins are recognized as superantigens [69], as is the staphylococcal toxic shock toxin [70], the Mls murine self-antigens [71], and Mycoplasma arthritidis mitogen [72]. They are distinguished not only by their characteristic binding, but also by the ensuing induction of a potent T cell proliferation. SEB, as a superantigen, possesses the ability to bind directly to the α chain of major histocompatibility complex (MHC) class II glycoprotein [73], outside the peptide-binding groove of antigen presenting cells, effectively bypassing normal antigen processing and presenting mechanisms. However, the binding of some SAgs to MHC proteins can be distinguished by an additional zinc-mediated, higher-affinity binding site on the HLA DR β chain mediated by the conserved H81 histidine residue [74,75]. This interaction has been reported with SEA, SED, SEE, SEH [74,75,76], and can result in a crosslinking of the MHC on the surface of antigen-presenting cells [77]. The molecular interface of SEB with the αβT cell receptor occurs by SEB’s attachment to the external Vβ domain in the TCR, resulting in the stimulation of a large proportion of CD4^+^ and CD8^+^ T cells (5%–30%) without regard to antigenic specificity or repertoire [27,31,78]. Thus, bound SEB interfaces with 5 residues (Tyr50, Ala52, Gly53, Thr55, and Ser54) within the complementarity determining region 2 (CDR2) of the TCR Vβ, 5 residues within FR3 (Ala67, Lys66, Tyr65, Lys57, Glu56), and to a lesser extent, two residues (Pro70, Ser71) within hypervariable region 4 (HV4), and a single His47 residue within framework region 2 (FR2). In contrast, the binding sites for SEC3 include 6 residues within CDR2 (Tyr50, Gly51, Ala52, Gly53, Ser54, Thr55), 3 residues within FR3 (Glu56, Lys57, Lys66), and 2 residues (Pro70, Ser71) within HV4 [79]. Significantly, these differences demonstrate that the specificity of the SAg binding to the TCR is primarily determined by complementarity determining region 2 (CDR2) of the TCR Vβ chain, and framework region 3 (FR3). The activated T cells proliferate and release massive proinflammatory cytokines that can lead to the development of clinical symptoms such as fever, swollen lymph nodes. Critically, the immunotoxic effects of the SEs are ultimately dependent upon the presence of reactive T lymphocytes. Thus, T cell-deficient nude mice have been shown to be protected from SE-induced shock, weight loss, and death [80]. Severe combined immunodeficiency (SCID) mice, which are deficient in T and B cells, appear to be resistant to the enteropathic effects of SEB. In this instance, intraperitoneal administration of SEB into either immunologically intact normal Balb/c mice or reconstituted SCID mice induces a jejunal histopathology characterized by reduced villus height, increased crypt depth, and upregulated MHC class II expression. However, the SE-induced enteropathy can only be achieved when the SCID mice are reconstituted with either mixed lymphocytes or with CD4^+^ cells [81,82].

## 3. Immunopathology of the Staphylococcal enterotoxins within the Intestinal Mucosa

**Figure 1:**
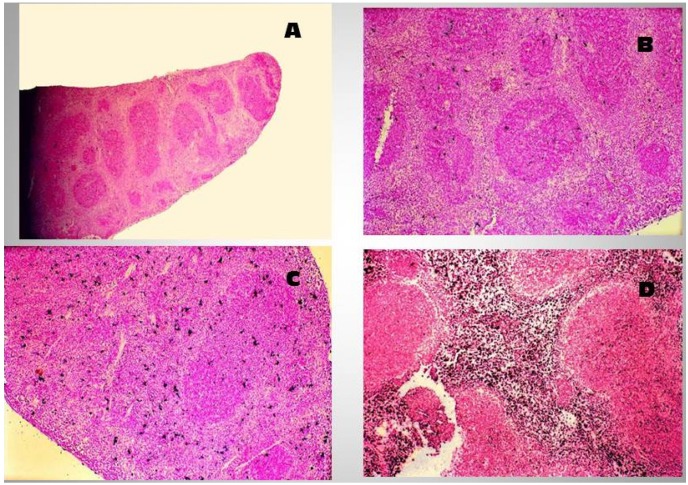
Staphylococcal enterotoxin B (SEB) induces apoptosis in mouse Spleen. (**A**) Normal splenic tissue from a 12 week-old C57BL/10J mouse as seen in 10× magnification and 20× (**B**); Representative Peyer’s patch tissue excised from a 12 week-old C57BL/10J mouse gavaged with SEB is shown in 20× magnifcation (**C**) and 40× (**D**). Tissues were excised 6 days following oral treatment. Immunohistochemical TUNEL staining of the tissues was performed where darkly stained cells are indicative of apoptosis. Note the darkly-stained apoptotic cells in the expanded follicular area of the SEB-treated spleen section. Staining was performed using a Trevigen TACS^R^ TdT In Situ Apoptosis Detection Kit.

**Figure 2:**
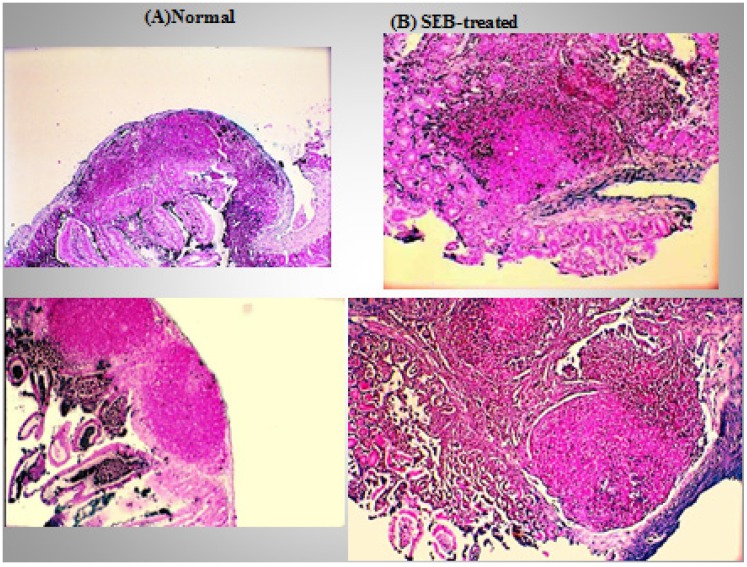
Staphylococcal enterotoxin B (SEB) induces apoptosis in mouse Peyer’s patches. (**A**) Normal Peyer’s patch tissue from a 12 week-old C57BL/10J mouse demonstrating germinal center containing lymphocytes; (**B**) Representative Peyer’s patch tissue excised from a 12 week-old C57BL/10J mouse gavaged with SEB. Tissues were excised 6 days following oral treatment. Immunohistochemical TUNEL staining of the tissues was performed where darkly stained cells are indicative of apoptosis. Note the darkly-stained apoptotic cells in the expanded follicular area of the SEB-treated Peyer’s patch section. Staining was performed using a Trevigen TACS^R^ TdT In Situ Apoptosis Detection Kit.

When ingested, SEs of the classic serotypes function both as potent gastrointestinal toxins and superantigens; however, they also possess the ability to traverse the intact intestinal epithelium. This was demonstrated using a mouse model using B10.BR mice that were inoculated orally with either SEA or SEB. Blood serum obtained from these animals induced IL2 production in T hybridomas bearing the appropriate surface TCR, providing strong evidence that the toxin(s) readily crossed the intact intestinal epithelium. Using an *in vitro* culture system, Hamad and coworkers further showed that MHC class II-negative human intestinal epithelial cells (Caco-2) can transcytose SEB and other bacterial superantigenic toxins in a dose-dependent manner [83]. Interestingly, mutated SEB containing either a mutation in the TCR biding site (N23) or a mutation in the area that binds the MHC class II molecule (F44) were weak stimulators of T cell proliferation [84]. In their study, Hamad and coworkers demonstrated a marked reduction in transcytosis, indicating the importance of these residues in transcytosis [83]. SEB exposure has also been shown to reduce the expression of mucosal tight-junction and adherent-junction proteins, leading to increased permeability and intestinal secretion [85,86]. The disturbance of the intestinal epithelial tight junction is probably associated with the enhanced secretion of IFN-γ and tumor necrosis factor (TNF) from lymphocytes. SEB treatment of rabbit intestinal segments instigates a rapid and large amount of structural destruction, and the damaging effect of SEB appeared to be more prominent in the small, rather than in the large, intestine. The primary target of the toxin was the epithelial cells along the length of the villi, followed by the lamina propria [87]. When ingested, SEs may cause emesis, exaggerated intestinal peristalsis, and pronounced alteration in **i**ntestinal mucosa structure when administered enterally in monkeys, dogs, or pigs [27]. The extent of the response reflects the toxin’s superantigenic capacity, by which SEs activate gastrointestinal T cells and provoke a “cytokine storm”. The numerous cytokines released include Th1 (IL-2 and IFN-γ), Th2 (IL-4), and Th17 (IL-17) cytokines from CD4^+^ T lymphocytes as well as IL-1β, IL-6, IL-8, and TNF from activated macrophages and other cell types. These cytokines may act as chemoattractants and induce expression of adhesion molecules, favoring localization of diverse immune cells responding to SE challenge. Notably, distinct cytokines stimulate functional maturation of immune cells and boost their response to SEs. In addition, many cytokines can affect intestinal epithelial cell functions, particularly ion and water transport. Early research in which rats were fed, portrayed the striking events induced by the toxin within the gastrointestinal tract; specifically, a rapid inflammatory response, confined primarily to stomach and duodenum [88]. The upper gastrointestinal tract of the challenged rats displayed a marked infiltration of different types of inflammatory cells in the epithelium and lamina propria, with some evidence of subepithelial edema and watery exudate containing cells and mucus. In a mouse model, intragastric administration of SEB has been shown to induce an early activation and expansion of responsive Vβ8^+^ T cells in PP and MLN [89]. RT-PCR analysis of cytokine mRNA in purified Vβ8^+^ T cells showed that SEB significantly upregulated the mRNA expression of IL-2 and IFN-γ. In the MLN, mRNA specific for IL-2 was increased by 100-fold and IFN-γ mRNA was increased 20-fold. In the spleen, mRNA for IL-2 was increased 10-fold, while IFN-γ was increased 5-fold. Data derived from this laboratory has demonstrated that ingestion of SEB by C57Bl/10J mice can result in a dose-dependent induction of hyperplastic proliferation in peripheral lymphoid organs such as the spleen (Figure 1), resulting in a hyperplastic proliferation that alters the normal follicular architecture in spleen and Peyer’s Patches (Figure 2) by 6 days after ingestion of the toxin [90] and a marked increase in apoptotic events as evidenced in both the spleen and Peyer’s Patches of treated mice when compared to normals. Further, ingestion of the toxin will also induce dramatic changes with respect to the localization of B220^+^ cells in the PP of the gastrointestinal tract [91]. In other research, intraperitoneal administration of SEB has triggered enterocolitis in mice [81,82] and rats [85,92,93], marked by intestinal recruitment of innate immune cells, activation of T helper lymphocytes, and enhanced release of TNF and IFN-γ. Intraepithelial lymphocytes are thought to play an important role in the pathophysiologic response to Staphylococcal infection. SEB challenge has also been shown to increase the γδ T lymphocyte population in PP, lamina propria, and epithelium [92]. Further, in the presence of SEB, human jejunal IEL have exhibited an enhanced *in vitro* cytotoxic effect on human C1R B-lymphoblastoid cells and IFN-γ pretreated colonic adenocarcinoma cell clone (HT-29). This response was greater than what is induced by using IEL that were activated with either phytohaemagglutinin (PHA)-, IL-2-, or anti-TCR antibody. In this report, the enhanced cytotoxicity demonstrated by SEB-treated IEL, did not engage MHC class II, TCR, or CD1d [94]. 

## 4. Staphylococcal enterotoxins and Immune Tolerance

Immune tolerance demonstrates a specific suppression of cellular or humoral responses that can be induced by repeated administration of an antigen, either at very large doses or at small doses that are below the stimulation threshold [67]. The two primary mechanisms that account for tolerance are clonal deletion (anergy) of antigen-specific effector cells and active suppression by regulatory T cells [95,96,97]. Oral tolerance is one of the most important forms of the induced tolerance, which is driven by prior administration of antigen by the oral route and presumably evolved to prevent destructive reactivity to normally occurring food proteins and commensal bacterial antigens in the intestinal mucosa. Multiple factors may contribute to the development of tolerance, including antigen properties, route of exposure, and host susceptibility and age. However, the antigen’s dose is critical to the development of tolerance [98]. Larger doses of antigens lead to anergy [99] or deletion [97] of antigen-specific T cell clones, while induction of tolerance by small doses is mediated by regulatory cells (T reg). *In vivo* exposure of mice to SEs is has been implicated in inducing peripheral T cell tolerance. Mucosal introduction (by feeding) of SEA to neonatal mice has been shown to support the development of oral tolerance to OVA protein [100]. In these experiments, neonatal mice were exposed to SEA and fed OVA as adults, and intranasally challenged. Significantly, the animals demonstrated reduced production of serum IgE antibodies to the ingested challenge antigen and reduced inflammatory processes within the lungs. Other researchers have shown that oral administration of SEA and myelin basic protein results in an increase in Treg populations and IL-10 production [101]. 

Mice injected with single dose of SEA demonstrate a rapid production of the Th1 proinflammatory cytokines IL-2, TNF, and IFN-γ, and serum IL-10 is not detectable. Repeated SEA challenges generated high levels of IL-10, but the production of IL-2, TNF, and IFN-γ was impaired and gradually eliminated. Interestingly, pretreatment of the mice with neutralizing anti-IL-10 mAb before the SEA challenges resulted in an increased IFN-γ and TNF response [102]. When stimulated with peptides *in vitro*, spleen cells from SEA-treated mice exhibited profound blocks in proliferation and IL-2 responses as compared to naïve spleen cells. Both SEA-treated CD4^+^ cells and supernatants from cultures of stimulated SEA-treated spleen cells had the capacity to inhibit IL-2 production by stimulated naïve spleen cells [103]. The supernatant-mediated suppression could be blocked by addition of the antibodies against IL-10 and TGF-β. However, only anti-TGF-γ antibody could partially restore the IL-2 production in co-cultures with the SEA-treated spleen cells, suggesting that efficient suppression of the co-cultured primary-stimulated spleen cells might be largely mediated by other mechanisms not involving IL-10 and TGF-β, such as the direct contact of primary effector cells with the regulatory cells within the SEA-treated cell population. 

Repeated SEA or SEB exposure may induce anergy and regulatory T cell function in mice. The *in vivo*-tolerized CD4^+^ T cells are functionally similar to the natural Tregs, expressing elevated levels of CTLA and suppressing T cell proliferation [104,105]. Continuous stimulation of human CD4^+^CD25^−^ cells or CD8^+^CD25^−^ cells with SEB *in vitro* also leads to generation of adaptive Foxp3-expressing T cells with potent immuno-suppressive properties [106,107]. Spleen cells from mice made tolerant to SEA and/or SEB by repeated injection of the toxins were able to transfer their state of unresponsiveness to naive syngeneic recipient mice *in vivo* and to primary-stimulated T cells *in vitro* [108]. The production of IL-2 and IFN-γ following SE stimulation was greatly impaired in the animals adoptively transferred with the unresponsive spleen cells. Similarly, in the presence of CD4^+^ T cells from the SE-tolerant animals, the primary-stimulated T cells showed significantly reduced cytokine responses to SE *in vitro* [108]. The mechanism of the regulatory function of the SE-unresponsive cells likely involves the expression of CTLA and the secretion of IL-10 [109]. Further study demonstrated that in mice made unresponsive to SEB after chronic exposure to low doses of the toxin, the reactive CD4^+^TCR-Vβ8^+^ T cells with regulatory function contained two subpopulations, *i.e.*, CD4^+^CD25^+^ Treg and CD4^+^CD25^−^ T cells [110]. The Treg cells control the induction phase of the tolerant state and control primary SEB-induced T cell proliferation. The tolerant state could not be reached in thymectomized CD25^+^ cell-depleted mice and repeated injection of SEB was unable to protect these animals from lethal toxic shock. In contrast, CD4^+^CD25^−^ T regulatory cells which did not express Foxp3 and CTLA, have been shown to exert a regulatory effect upon the SEB response. Namely, addition of purified CD4^+^CD25^−^ from SEB-tolerant mice to primary cultures of normal spleen cells cultured with SEB resulted in a reduction of SEB-induced proliferation. These results indicate the regulatory role of the CD4^+^CD25^−^ cells in the maintenance of the SEB tolerant state. However, a recent study from Eroukhmanoff *et al.* argued that repeated immunization with SEB did not really upregulate Foxp3 expression. The increased frequency of Foxp3^+^ cells in spleen and MLN of immune tolerant mice was actually due to a reduced number of antigen-reactive conventional CD4^+^ T cells rather than the conversion of these cells to Foxp3^+^ Treg [111]. Breakdown of oral tolerance may lead to the development of some autoimmune diseases, some of which may include inflammatory bowel disease processes, such as ulcerative colitis and Crohn’s disease, and celiac disease.

## 5. Staphylococcal enterotoxins in Inflammatory Bowel Diseases

The etiologies of inflammatory bowel diseases such as ulcerative colitis and Crohn’s disease remain unclear but an uncontrolled T cell-mediated immune response to non-pathogenic commensal luminal bacteria in a genetically susceptible host has been proposed [112]. A growing body of evidence suggests that the SEs are implicated in the pathogenesis of inflammatory bowel diseases. A subgroup of Crohn’s disease patients display an increased number of Vβ8-expressing T cells, both CD4^+^ and CD8^+^, in peripheral blood and MLN [113]. A significant over-expression of Vβ5.1 and Vβ8 gene segments was also determined after long-term cultivation of colonic biopsies from Crohn’s disease patients *versus* controls [114]. In that study, the cultures were set up under conditions that favor T cell growth. Since neither feeder cells nor any exogenous antigens that may modify Vβ gene expression were applied, T cell activation was solely dependent on biopsy-derived endogenous stimulants and the results indicated a prior *in vivo* exposure to Vβ8-selective superantigens. Indeed, *in vitro* stimulation of colonic explants from patients with Crohn’s disease or ulcerative colitis with SEB or SEE led to the expansion of T cells expressing Vβ8 and a concomitant inflammatory cytokine release; the response was greater in the inflamed than non-inflamed tissues [114,115,116]. Interestingly, the cytotoxic function of Vβ8^+^ cells seemed to be compromised in Crohn’s disease patients [115]. While Vβ8^+^ T cells seem to play a role in Crohn’s disease, selective expansion of T cells bearing TCR-Vβ4 has been demonstrated in patients with ulcerative colitis. Patients with skewed TCR-Vβ4-expressing cells are reported to have longer disease duration as compared with patients with low level of TCR-Vβ4 [117]. In this instance, Streptococcal mitogenic exotoxin Z-2 (SMEZ-2), produced by Streptococcus pyogene, is known to preferentially activate the TCR-Vβ4-bearing T cells. Patients with skewed TCR-Vβ4demonstrate significantly higher level of anti-SMEZ-2 antibodies, suggesting an intimate relationship between the bacterial toxin and the bowel disease status. Another pathway for the induction of inflammatory bowel disease has been associated with pre-existing chronic rhinosinusitis by a mechanism of swallowing sinusitis-derived SEB. Early studies documented an increased frequency of enterotoxigenic *Staphylococcus aureus* in the nares of individuals diagnosed with allergic rhinitis [118]. Patients with both ulcerative colitis and chronic rhinosinusitis present significant sinus infection with *Staphylococcus aureus*, accompanied by high levels of SEB in the sinus wash fluids, anti-SEB antibody in the sera, and anti-SEB positive-cells in the colonic mucosa [7]. After functional endoscopic sinus surgery for rhinosinusitis, these patients showed ameliorated intestinal inflammation. Bacteriologic analysis of removed sinus mucosal specimens showed that the colony number of cultured *Staphylococcus aureus* correlated with the decrease in disease severity of ulcerative colitis. When cultured *in vitro* with SEB, colonic biopsy-derived mast cells from the patients with both ulcerative colitis and chronic rhinosinusitis demonstrated massive degranulation and release of histamine and tryptase, as compared to the patients with ulcerative colitis only, or the healthy controls. Similarly, introducing sinusitis-derived SEB to murine gastrointestinal tract also caused increased colonic epithelial permeability and impaired mucosal barrier function [119]. Mice sensitized by intra-gastric gavage of ovalbumin (OVA) in the presence of SEB-containing sinus wash fluid from patients with chronic rhinosinusitis and then challenged with OVA, developed mucosal immunopathology in the colon, evidenced by marked degranulation of mast cells and eosinophils, infiltration of inflammatory cells, mucosal ulceration, and abscess formation in the lamina propria. In contrast, the inflammatory state was not induced in the mice sensitized to OVA only or when anti-SEB antibody was added to the sensitizing mixture. Likewise, intra-rectal administration of SEA and SEB resulted in colonic inflammation in a time- and dose-dependent manner. Moreover this treatment aggravated the illness in mice recovering from dextran-sodium sulfate-induced colitis [120]. In a SCID mouse model of colitis, feeding SEB to mice reconstituted with CD4^+^CD45RB^high^ T cells resulted in an earlier onset of intestinal inflammation and more severe symptoms, which was accompanied by activation and expansion of SEB-reactive CD4^+^Vβ8^+^ T cells and marked impairment in CD4^+^CD25^+^Foxp3^+^ Treg cell development . 

## 6. Conclusions

The SEs exert remarkable responsiveness in the gastrointestinal tract where they provoke diverse innate and adaptive immune events involving both exaggerated inflammatory and tolerant states. SEs are not only incriminated in food poisoning episodes but are also implicated in various inflammatory and autoimmune conditions such as those reviewed in this paper. That the toxins are able to exert such a diversity of activities in the digestive tract is extraordinary, but is evidently associated with the unique local microenvironment**.** Despite intensive efforts, the nature of the intertwined interaction between SEs and the different varieties of cells, cytokines, signaling molecules, and transcription factors in intestinal mucosa is yet to be fully understood. The precise mechanisms that trigger the inflammatory or tolerance processes are fundamentally unknown. Although it remains to be established whether targeting SEs, either to block their superantigenicity or to use as a tool to generate immune tolerance to disease-inducing proteins, is appropriate or adequate, this approach has the potential to provide a therapeutic benefit in human food-borne and immune-based gastrointestinal diseases.

## Declare

This article reflects the views of the authors and should not be construed to represent views or policies of the U.S. Food and Drug Administration.
